# Assessment of psychological and physical stressors among nurses in different functional areas before and during the COVID-19 pandemic: a cross-sectional study

**DOI:** 10.1186/s12912-023-01424-4

**Published:** 2023-08-07

**Authors:** Philipp Winnand, Yvonne Fait, Mark Ooms, Anna Bock, Marius Heitzer, Thea Laurentius, Leo Cornelius Bollheimer, Frank Hölzle, Janosch A. Priebe, Ali Modabber

**Affiliations:** 1 Department of Oral and Maxillofacial Surgery, University Hospital RWTH Aachen, Pauwelsstraße 30, D-52074 Aachen, Germany; 2 Department of Geriatrics, University Hospital RWTH Aachen, Pauwelsstraße 30, D-52074 Aachen, Germany; 3 Department of Neurology, Center for Interdisciplinary Pain Management-Rise-uP, Klinikum rechts der Isar, MRI, Technical University of Munich, Ismaninger Str. 22, D-81675 Munich, Germany

**Keywords:** Burnout, COVID-19 pandemic, Nursing, Resilience, Stress

## Abstract

**Background:**

The COVID-19 (coronavirus disease) pandemic placed a great burden on all health-care resources, especially nurses. The prevalence and underlying risk factors of affective symptoms related to the COVID-19 pandemic have been studied primarily among nurses in intensive care units (ICU) and emergency departments. The aim of this study was to identify at-risk nursing areas by examining the psychological and physical stress values of nurses in different functional areas.

**Methods:**

A questionnaire with standardized items was developed to assess psychological and physical stress values. At least 50 nurses with a minimum work experience of 3 years were recruited from the ward, outpatient clinic (OC), intermediate care (IMC) unit, and operating room (OR) of the University Hospital RWTH Aachen. The participants answered the questionnaire by referring to their perceptions before and during the COVID-19 pandemic. Absolute differences and relative trends in psychological and physical stress values were compared within and across functional areas.

**Results:**

The ward and OR nurses experienced significant increases in workload (p < 0.001 and p = 0.004, respectively) and time stressors (p < 0.001 and p = 0.043, respectively) during the COVID-19 pandemic. Regardless of functional area, the nurses showed strong tendencies toward increases in subclinical affective symptoms. After adjustments for age, sex, working in a shift system, the treatment of patients with COVID-19, and the impact of the COVID-19 pandemic on personal life, the values for working with pleasure decreased significantly among the ward (p = 0.001) and OR nurses (p = 0.009) compared with the OC nurses. In addition, the ward (p < 0.001) and OR nurses (p = 0.024) were significantly more likely to express intent to leave their profession than OC nurses.

**Conclusions:**

The IMC nurses showed good adaptation to the exacerbated situation caused by the COVID-19 pandemic. The ward nurses, followed by the OR nurses, were the most vulnerable to mental and physical exhaustion, which threatened the nurses’ resilience and retention in the wake of the COVID-19 pandemic. Therefore, intervention programs must specifically address the professional and emotional needs of ward and OR nurses to prepare the health-care system for future crises.

## Background

The outbreak of a novel coronavirus variant in Wuhan, China, in December 2019 [1] was designated COVID-19 (coronavirus disease) by the World Health Organization (WHO) and eventually declared a pandemic in March 2020 after its rapid global spread [2]. The pandemic outbreak placed a high burden on the resilience of all health-care resources [3], including public health functions, medical products and technologies, critical care treatment capacities and health workforce [4, 5]. Frontline nurses were exposed to high health risks through direct care and treatment of patients with COVID-19 [6].

The overload of the health-care system, caught unprepared by the COVID-19 crisis, led to sharp increases in nurses’ workload, work complexity, work pressure, and work hours [7]. Even more so than physicians, nurses showed a superior increase in affective symptoms as a result of the increased physical stress caused by the COVID-19 pandemic [8–10]. Against this background, nurses continue to be at risk of feeling emotionally exhausted, depersonalized, and personally unfulfilled [7], which translates into stress, depression, and burnout [11].

The prevalence of burnout and its underlying risk factors have been extensively studied among nurses in intensive care units (ICU) and emergency departments [12–15], with sociodemographic, social, and occupational factors identified as critical contributors to burnout disorders. The development of burnout among nurses often results in an intention to leave the profession [16], which negatively impacts patient care [17–19] and, as a result of temporary substitution, places a financial burden on the health-care system [20]. As the intention to leave the profession increases with nurses’ work experience, the health care workforce faces a steady loss of knowledge and experience [21–23]. The importance of team relationships in nursing is reflected in the promotion of mental health through good team relationships [7, 24] and the weakening of professional retention through poor relationships [25, 26].

The identification of nurses at risk and functional coping strategies can enhance nurses’ professional quality of life [27], which requires readiness for intervention by policy makers, hospital facilities, and individuals [28]. However, individualized coping strategies tailored to the specific needs of nurses are difficult to implement, as few studies have elucidated the physical and emotional stress characteristics of nurses in functional areas other than critical care and emergency nursing. Therefore, the aim of this study was to identify nursing areas at risk by examining the psychological and physical stress of nurses in different functional areas such as the ward, outpatient clinic (OC), intermediate care (IMC) unit, and operating room (OR) before and during the COVID-19 pandemic in a German maximum care hospital.

## Methods

### Study population and sample size calculation

The study was approved by the ethics committee of the Medical Faculty of RWTH Aachen University, Germany (EK 22–304). G*Power 3.1.9.7 [29] was used for sample size calculation. A significance level (α) of 5% and a power of 80% were assumed. In the absence of comparable studies for effect size, a medium Cohen’s f effect size of 0.25 was used [30]. With these parameters, a minimum sample size of 180 was specified. Because of the presence of nonparametric data, the calculated minimum sample size was increased by 15% [31] to 207.

Nurses were recruited from the ward, OC, IMC unit, and OR. For each functional area, at least 50 nurses were included. The demographic and clinical characteristics of the study population are listed in Table 1.

**Table 1:** Demographic and clinical characteristics of the study population

*Variable*	*Ward (n = 56)*	*OC (n = 54)*	*IMC (n = 64)*	*OR (n = 50)*	*p value*
***Age (years)***	45.0 (20)	42.0 (27)	36.0 (15)	41.0 (26)	**0.026**
***Sex (n)***					**< 0.001**
Male	8 (14.3%)	8 (14.8%)	27 (42.2%)	18 (36.0%)	
Female	48 (85.7%)	46 (85.2%)	37 (57.8%)	32 (64.0%)	
***Work experience (years)***	16.0 (25)	20.5 (28)	13.0 (18)	11.5 (25)	0.103
***Shift system (n)***					**< 0.001**
No	15 (26.8%)	45 (83.3%)	5 (7.8%)	25 (50.0%)	
Yes	41 (73.2%)	9 (16.7%)	59 (92.2%)	25 (50.0%)	
***Full-time employment (n)***					0.106
No	19 (33.9%)	14 (25.9%)	30 (46.9%)	16 (32.0%)	
Yes	37 (66.1%)	40 (74.1%)	34 (53.1%)	34 (68.0%)	
***Fear of contracting COVID-19 (n)***	2.0 (4)	1.0 (4)	2.0 (5)	2.0 (5)	0.313
***Impact of COVID-19 on personal life (n)***	2.0 (5)	1.0 (4)	3.0 (4)	2.0 (4)	**0.019**
***Treatment of patients with COVID-19 (n)***					**< 0.001**
No	11 (19.6%)	24 (44.4%)	3 (4.7%)	17 (34.0%)	
Yes	45 (80.4%)	30 (55.6%)	61 (95.3%)	33 (66.0%)	

Parameters are indicated as numbers (with percentages) or median values (with interquartile ranges) and separately described for nurses in the ward, outpatient clinic (OC), intermediate care (IMC) unit, and operating room (OR). Differences between the groups were analyzed using the chi-square test (sex, working in a shift system, full-time employment, and treatment of patients with COVID-19) or the Kruskal-Wallis test (age, work experience, fear of contracting COVID-19, and impact of COVID-19 on personal life). Significant p values are bold.

### Study design

The nurses were given a self-administered questionnaire, which queried demographic data and 24 items. The items were answered from pre-pandemic and current (during the COVID-19 pandemic) perspectives. The data were collected in December 2022. A minimum work experience of 3 years was considered an inclusion criterion and ensured that each participating nurse could also answer the questionnaire from a pre-pandemic perspective.

First, four self-constructed items on topics related to the COVID-19 pandemic were queried. Subsequently, subjective job satisfaction and stress were assessed using nine selected questions from the questionnaire developed by Weyer et al. [32]. Individual burnout risk was quantified using eleven selected questions from the three subscales (emotional exhaustion, personal accomplishment, and depersonalization) of the Maslach-Burnout Inventory (MBI), which typically uses 22 items on a 7-point frequency scale and sums the score for each subscale separately [33]. The questionnaires containing the items used in this study to query psychological and physical stressors related to the COVID-19 pandemic have been published elsewhere [32, 34]. With the exception of the demographic data query, all items in this study’s questionnaire were scored using a visual analog scale (VAS) with a minimum mark of 0 and a maximum mark of 10. The scores were exported to an MS Excel spreadsheet for further statistical analysis.

### Data collection

Participant recruitment and data collection were performed by a study nurse (YF) who personally visited the different functional nursing areas in compliance with COVID-19 regulations. The procedure and purpose of the study were explained in detail to the nurses. Nurses were then asked to complete the questionnaire anonymously. The questionnaire was answered voluntarily and took 5 to 10 min to complete. A total of 320 questionnaires were distributed, of which 224 were completed in full. All completed questionnaires were included for study analysis, exceeding the minimum sample size of 207.

### Statistical analysis

Baseline characteristics were indicated as numbers (with percentages) or median values (with interquartile ranges) and separately described for nurses at the ward, OC, IMC unit, and OR. Differences between the groups were analyzed using the chi-square test for categorical data (sex, working in a shift system, full-time employment, and treatment of patients with COVID-19) or the Kruskal-Wallis test for metrical data (age, work experience, fear of contracting COVID-19, and impact of COVID-19 on personal life).

The questions were summarized into clusters (disillusion, work gratification and stressors, exhaustion, working environment, and job satisfaction). Question scores were presented as median values (with interquartile ranges) and separately described for nurses in the ward, OC, IMC unit, and OR. Absolute differences within and between the groups were analyzed using the Wilcoxon signed-rank and Kruskal-Wallis tests, respectively. Dunn’s post hoc test was used for pairwise comparisons.

Relative trends were presented as numbers of nurses (with percentages) with higher or lower question scores (trend up = scores > 1; trend down = scores < 1). Differences in relative trends between all groups were analyzed using the Kruskal-Wallis test, whereas Dunn’s post hoc test was used for pairwise comparisons.

The relative trends between and within the groups were adjusted for age, sex, working in a shift system, the treatment of patients with COVID-19, and the impact of COVID-19 on personal life using logistic regression analysis. Statistical significance was assumed at p values < 0.05. Statistical analysis was performed using SPSS version 28 (SPSS, IBM, New York, NY). The pairwise comparison of the relative trends was visualized with GraphPad Prism version 9 (GraphPad Software, San Diego, CA).

## Results

### Demographic and clinical characteristics of the study population

The groups significantly differed in age (p = 0.026), sex (p < 0.001), and working in a shift system (p < 0.001). Work experience (p = 0.103), full-time employment (p = 0.106), and fear of contracting COVID-19 (p = 0.313) did not differ significantly between the groups. The impact of the COVID-19 pandemic on personal life (p = 0.019) and the treatment of patients with COVID-19 (p < 0.001) differed significantly between the groups (Table 1).

### Comparison within the groups

Compared with the pre-pandemic perception, the nurses’ perception at the end of the observation period indicated significantly increased feelings of frustration (ward: p < 0.001; OC: p = 0.028; IMC: p = 0.004; and OR: p = 0.026) and burnout (ward: p < 0.001; OC: p = 0.002; IMC: p = 0.039; and OR: p = 0.002) within the groups. Professional fulfillment was unchanged, with significantly increased time stress (ward: p < 0.001 and OR: p = 0.043) and workload (ward: p < 0.001 and OR: p = 0.004) among the ward and OR nurses. Emotional exhaustion significantly increased among the ward (p = 0.004) and OR nurses (p = 0.003) during the COVID-19 pandemic. Physical exhaustion was significantly increased among the ward (p < 0.001), OC (p = 0.02), and OR nurses (p = 0.006).

The relationship between colleagues was not affected by the COVID-19 pandemic. Mutual blaming increased significantly among the nurses in the ward (p = 0.002) and OR (p = 0.005), and workplace atmosphere deteriorated significantly in these areas (ward: p = 0.003 and OR: p < 0.001).

Working with pleasure decreased significantly among the OR nurses (p = 0.001). The ward (p < 0.001) and OR nurses (p = 0.009) were significantly more likely to consider leaving the profession (Table 2).

**Table 2:** Comparison of absolute differences within and between the groups

*Variable*		*Time*		*Ward (n = 56)*		*OC (n = 54)*		*IMC (n = 64)*		*OR (n = 50)*		*p value*
***Disillusion***												
Frustration		Pre		3.5 (5)^2, 3^		2.0 (4)		4.0 (5)^4^		3.0 (5)^4^		**0.013**
		Post		*6.0 (6)^1^		*2.0 (5)^2, 3, 4^		*5.5 (6)^1^		*4.5 (6)^1^		**0.002**
Burnout		Pre		4.0 (5)^3^		2.5 (5)		4.0 (4)^3^		3.0 (7)^2, 4^		**0.039**
		Post		*6.0 (6)		*4.0 (6)		*5.0 (5)		*4.0 (8)		0.053
***Work gratification and stressors***												
Professional fulfillment		Pre		8.0 (3)		7.0 (4)		7.0 (4)		7.0 (4)		0.268
		Post		7.5 (4)		7.0 (4)		7.0 (4)		7.0 (4)		0.610
Time pressure		Pre		6.0 (5)^1, 3^		5.0 (5)^4^		7.0 (6)		5.0 (4)^4^		**0.007**
		Post		*8.0 (5)^1^		5.0 (5)^2, 4^		7.5 (5)^1^		*5.5 (6)		**< 0.001**
Work overload		Pre		6.0 (6)^1^		5.0 (4)^2, 4^		8.0 (4)^1^		6.0 (5)		**0.002**
		Post		*8.0 (4)^1^		5.0 (4)^2, 4^		8.0 (4)^1^		*7.0 (6)		**< 0.001**
***Exhaustion***												
Emotional		Pre		5.0 (5)^1^		4.0 (5)^2, 4^		6.0 (5)^1^		4.0 (7)		**0.024**
		Post		*6.5 (5)		5.0 (5)^2^		6.0 (5)^1, 3^		*5.0 (6)^2^		**0.039**
Physical		Pre		8.0 (5)		7.0 (3)		8.0 (4)		6.5 (4)		0.234
		Post		*9.0 (2)		*8.0 (4)		8.0 (3)		*8.0 (4)		**0.008**
***Working environment***												
Relationship between colleagues		Pre		8.0 (2)		9.0 (4)		8.0 (2)		8.0 (3)		0.590
		Post		8.0 (4)		9.0 (3)		8.0 (2)		8.0 (3)		0.295
Mutual blaming		Pre		1.0 (3)		0.0 (1)		1.0 (3)		1.5 (3)		**0.048**
		Post		*1.0 (5)		0.0 (1)^3^		1.0 (3)		*2.0 (6)^1^		**0.013**
Workplace atmosphere		Pre		7.0 (4)		6.5 (4)		6.0 (3)		6.0 (4)		0.934
		Post		*6.0 (4)		7.0 (4)		6.5 (3)		*5.0 (4)		0.105
***Job satisfaction***												
Work with pleasure		Pre		7.5 (4)^1^		8.0 (4)^2, 4^		6.0 (3)^1^		8.0 (3)		**0.015**
		Post		6.0 (3)		8.0 (4)		6.0 (3)		*7.0 (5)		0.099
Intent to leave the profession		Pre		2.0 (7)		0.5 (5)		3.0 (6)		2.5 (5)		0.103
		Post		*4.5 (8)		1.0 (5)^2^		4.0 (7)^1^		*4.0 (8)		**0.041**

Parameters are indicated as median values (with interquartile ranges) and separately described for nurses in the ward, outpatient clinic (OC), intermediate care (IMC) unit, and operating room (OR). Differences within and between the groups were analyzed using the Wilcoxon signed-rank and Kruskal-Wallis tests, respectively. Significant p values are bold.

*p < 0.05 vs. pre-pandemic question score within the groups.

1: p < 0.05 vs. OC.

2: p < 0.05 vs. IMC unit.

3: p < 0.05 vs. OR.

4: p < 0.05 vs. ward.

### Comparison between the groups

Before the onset of the COVID-19 pandemic, burnout (p = 0.039) and working with pleasure (p = 0.015) were significantly different between the groups. During the COVID-19 pandemic, physical exhaustion (p = 0.008) and the intent to leave the profession (p = 0.041) significantly differed between the groups. Before and during the COVID-19 pandemic, frustration (before and during, respectively: p = 0.013 and p = 0.002), time pressure (p = 0.007 and p < 0.001), work overload (p = 0.002 and p < 0.001), emotional exhaustion (p = 0.024 and p = 0.039), and mutual blaming (p = 0.048 and p = 0.013) were significantly different between the groups. The remaining items of the questionnaire did not differ between the groups (Table 2).

### Comparison of relative trends between the groups

With regard to the relative trends, work overload (p = 0.028), relationships between colleagues (p = 0.029), and workplace atmosphere (p = 0.025) were rated significantly differently across the functional areas. Before and after adjustments for age, sex, working in a shift system, the treatment of patients with COVID-19, and the impact of COVID-19 on personal life, the values for mutual blaming (p = 0.009), working with pleasure (p = 0.006), and intent to leave (p = 0.009) were rated significantly differently (Table 3).

**Table 3:** Comparison of relative trends between the groups

*Variable*	*Trend*	*Ward (n = 56)*	*OC (n = 54)*	*IMC (n = 64)*	*OR (n = 50)*	*p value*
**Disillusion**						
Frustration	Up	23 (41.1%)	13 (24.1%)	25 (39.1%)	12 (24.0%)	0.089
Burnout	Up	23 (41.1%)	19 (35.2%)	20 (31.3%)	16 (32.0%)	0.682
**Work gratification and stressors**						
Professional fulfillment	Down	8 (14.3%)	1 (1.9%)	10 (15.6%)	8 (16.0%)	0.070
Time pressure	Up	21 (37.5%)	8 (14.8%)	15 (23.4%)	12 (24.0%)	0.051
Work overload	Up	20 (35.7%)	6 (11.1%)	16 (25.0%)	12 (24.0%)	**0.028**
**Exhaustion**						
Emotional	Up	21 (37.5%)	18 (33.3%)	16 (25.0%)	16 (32.0%)	0.519
Physical	Up	23 (41.1%)	13 (24.1%)	14 (21.9%)	13 (26.0%)	0.092
**Working environment**						
Relationships between colleagues	Down	11 (19.6%)	1 (1.9%)	10 (15.6%)	9 (18.0%)	**0.029**
Mutual blaming	Up	10 (17.9%)	1 (1.9%)	5 (7.8%)	10 (20.0%)	**0.009** ^**+**^
Workplace atmosphere	Down	19 (33.9%)	7 (13.0%)	12 (18.8%)	16 (32.0%)	**0.025**
**Job satisfaction**						
Work with pleasure	Down	21 (37.5%)	6 (11.1%)	13 (20.3%)	16 (32.0%)	**0.006** ^**+**^
Intent to leave the profession	Up	20 (35.7%)	5 (9.3%)	13 (20.3%)	13 (26.0%)	**0.009** ^**+**^

Data are presented as numbers of nurses (with percentages) with higher or lower question scores (trend up = scores > 1; trend down = scores < 1) and separately described for nurses in the ward, outpatient clinic (OC), intermediate care (IMC) unit, and operating room (OR). Differences in relative trends between all groups were analyzed using the Kruskal-Wallis test. Significant p values are bold. ^**+**^after adjustments for age, sex, working in a shift system, treatment of patients with COVID-19, and impact of COVID-19 on personal life.

### Comparison of relative trends between two groups

Mutual blaming was significantly more common among the ward nurses than among the OC nurses (p = 0.005). After adjustments for age, sex, working in a shift system, the treatment of patients with COVID-19, and the impact of COVID-19 on personal life, mutual blaming was significantly more frequent among the OR nurses than among the OC nurses (p = 0.003).

Working with pleasure was significantly different between the ward and IMC nurses (p = 0.037). After adjustments for age, sex, working in a shift system, the treatment of patients with COVID-19, and the impact of COVID-19 on personal life, the pleasure of working in the ward (p = 0.001) and OR (p = 0.009) decreased significantly compared with the pleasure of working in the OC.

**Fig. 1:**
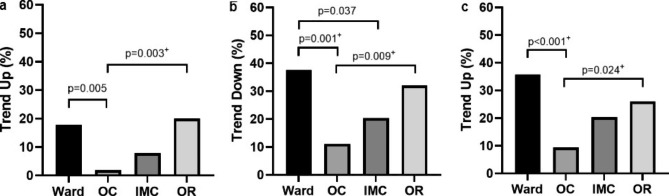
Pairwise comparison of relative trends. Relative trends of mutual blaming (a), working with pleasure (b), and intention to leave the profession (c) are presented as percentages of nurses with higher or lower question scores (trend up = scores > 1; trend down = scores < 1) and separately visualized for nurses in the ward, outpatient clinic (OC), intermediate care (IMC) unit, and operating room (OR). Pairwise testing of relative trends was performed for significant values of general testing between all groups (a: p = 0.009^**+**^; b: p = 0.006^**+**^; c: p = 0.009^**+**^). ^**+**^after adjustments for age, sex, working in a shift system, treatment of patients with COVID-19, and impact of COVID-19 on personal life

After adjustments for age, sex, working in a shift system, treatment of patients with COVID-19, and the impact of COVID-19 on personal life, the ward (p < 0.001) and OR nurses (p = 0.024) were significantly more likely to express an intent to leave the profession than the OC nurses (Table 4; Fig. 1).

**Table 4:** Pairwise comparison of relative trends

*Variable*	*Ward vs. OC*	*Ward vs. IMC*	*Ward vs. OR*	*OC vs. IMC*	*OC vs. OR*	*IMC vs. OR*
**Work environment**						
Mutual blaming	**0.005**	0.097	0.778	0.142	**0.003** ^**+**^	0.056
**Job satisfaction**						
Work with pleasure	**0.001** ^**+**^	**0.037**	0.553	0.175	**0.009** ^**+**^	0.155
Intent to leave the profession	**< 0.001** ^**+**^	0.059	0.281	0.096	**0.024** ^**+**^	0.473

Data are presented as p values corresponding to testing of two groups (ward vs. outpatient clinic (OC), ward vs. intermediate care (IMC) unit, ward vs. operating room (OR), OC vs. IMC unit, OC vs. OR, IMC unit vs. OR) using Dunn’s post hoc test for pairwise comparisons. Significant p values are bold. ^**+**^after adjustments for age, sex, working in a shift system, treatment of patients with COVID-19, and impact of COVID-19 on personal life.

## Discussion

### Summary of findings

In the wake of the COVID-19 pandemic, an increase in subclinical affective symptoms was observed in all nursing areas. However, the psychological and physical stressors were perceived differently by the nurses across functional areas. On the ward and in the OR, nurses suffered from increased workload and time stress. Ward and OR nurses experienced a deterioration of the workplace atmosphere with increased mutual blaming. In addition to the greatest loss of working with pleasure, OR and ward nurses most frequently expressed the intention to leave the profession.

### Burnout and frustration

Burnout threatens individual resilience among nurses [35], who are a critical workforce that enables the health-care system to function and respond to crises such as the COVID-19 pandemic.

Many studies have used the standardized, multi-item MBI to measure burnout in the three dimensions of emotional exhaustion, depersonalization, and personal accomplishment [7]. However, the cut-off values are inconsistent and not universally defined [36]. On the basis of the detection of early signs of burnout [37] and the accurate assessment of the emotional exhaustion subscale [38], single-item questions were alternatively suggested [39, 40]. In this study, the selected single items of the MBI [33] were used to assess subclinical affective symptoms and to quantify absolute differences and relative trends in the physical and psychological stress values of nurses from different functional areas.

One striking observation is that regardless of functional area, a non-negligible proportion (24–41%) of nurses showed increased feelings of frustration and burnout as a result of the COVID-19 pandemic. Assuming that frontline nurses in ICUs and emergency departments had the highest risk of burnout independently [41] and during the COVID-19 pandemic [7, 42], previous meta-analyses have estimated the pooled prevalence of the emotional exhaustion dimension of burnout to be 22.8% [43], 34.1% [7], and 42% [44]. Complementing the results reported in the existing literature, our data showed that ward, OC, and OR nurses, similar to IMC nurses, have high absolute scores and sharply increasing tendencies toward feeling frustrated and burned out, which is consistent with the findings of Wu et al. [45], who were the first to describe higher levels of burnout among nurses in normal wards with uninfected patients than in those in frontline wards with infected patients.

### Workload and stress

Workload is a significant factor of stress among nurses working in under-resourced health-care systems [46]. Prior to the COVID-19 pandemic, absolute workload and time pressure values were highest among nurses in the IMC unit. These perceptions did not change significantly among IMC nurses during the COVID-19 pandemic, which could be attributed to anticipatory measures to reduce ICU nurses’ workload, as intensive care beds were kept free for COVID-19 patients [47] and non-intensive care nurses were kept available for support in ICUs [48]. It is interesting that the IMC unit is characterized by the lowest median age of nurses and the highest proportion of male nurses compared with the other functional areas. While female sex has been associated with higher stress levels [49], the influence of nurses’ ages on their perceptions of stress related to the COVID-19 pandemic is controversial, with older [50] or younger age [11] as a risk factor.

The initial phase of the COVID-19 pandemic saw cancelations and, subsequently, a massive backlog of elective surgeries, which was addressed acutely by increasing surgical capacity [51], and sustainably by developing precautionary strategies [52]. The anticipated measures to restore hospital operations appeared to ignore the individual workload limits of nurses, mostly those in the normal wards, followed by those in the OR, which are the only functional areas characterized by significant increases in absolute workload and time pressure values. The relative trends showed that increasing stress levels appeared to have equal effects on nurses in the ward, IMC unit, and OR, but less effects on those in the OC.

The associations of workload and perceived stress with the work engagement [53] and mental health [54–56] of nurses can be traced in our collected data, as nurses in the wards and OR also experienced the greatest increases in absolute psychological and physical exhaustion levels. Although simultaneously occurring physical and psychological exhaustion has already been described for intensive care nurses in the context of the COVID-19 pandemic [50], their complex interrelationships cannot yet be explained in detail. A bidirectional interaction with mutual reinforcement between physical stress and psychological symptoms is suspected [57] but needs further investigation.

### Work environment conditions

Work environment conditions are critical factors that influence nurses’ mental health [58]. In general, the observation that relationships among colleagues did not change significantly as a result of the COVID-19 pandemic in all functional areas underscores the high protective value of social support for nurses’ mental health [7, 24]. After the synopsis of our findings revealed that nurses in the wards and OR were affected by high-intensity stressors and vulnerable to psychological and physical exhaustion, a significant deterioration of workplace atmosphere with increased mutual blaming was found exclusively among the ward and OR nurses in the wake of the COVID-19 pandemic.

As nurses’ perceptions of stress in the wake of the COVID-19 pandemic were influenced by demographic, social, and occupational factors [7], we adjusted the relative trends in psychological and physical stress values for any characteristics that differed significantly between the functional areas. After adjustments for age, sex, working in a shift system, the treatment of patients with COVID-19, and the impact of COVID-19 on personal life, relative trends of mutual blaming, working with pleasure, and intention to leave the profession continued to differ significantly between the functional areas. The ward and OR nurses showing the greatest loss of working with pleasure (37.5% and 32%, respectively) and the most frequent intention to leave the profession (35.7% and 26%, respectively) is an alarming signal but must be viewed as a logical consequence of the physical and psychological stressors described above. A meta-analysis revealed that 31.7% of nurses had an intention to leave the profession regardless of functional area [59], whereas 32.14% of OR nurses [60] and 23.4% of ward nurses [61] had an intention to leave the profession. In general, the high proportion of nurses with an intention to leave the profession in the wake of the COVID-19 pandemic appears to be an already known phenomenon. However, the high proportion of ward nurses with an intention to leave the profession may pose a threat to health-care system resilience, which has been underestimated in the literature to date.

### Strengths and limitations

The results of this study must be considered under minor limitations. Only nurses from one hospital facility were included in the questionnaire survey. As COVID-19 control measures were not implemented uniformly among German federal states [62], no statement can be made regarding the generalizability of the study results to other federal states and to Germany as a whole. The possible recall bias due to memory distortions could not be eliminated by answering the questionnaire once by referring to perceptions before and during the COVID-19 pandemic. To decisively prevent recall bias, the questionnaire should ideally have been answered at two time points in a longitudinal study design. With a questionnaire response rate of 70%, selection bias could not be excluded. This can be relevant if only nurses who felt or did not feel burdened were included in the survey. The timing of the collection of questionnaire responses when the COVID-19 pandemic was under control in Germany might have influenced the results of the present study. However, against the background of persistently high burnout rates in times of low COVID-19 incidence rates [28], this seems to be a negligible factor.

The design of our questionnaire, which consisted of individual items selected from standardized questionnaires by Weyer et al. [32] and Maslach et al. [33], limits comparability with other studies. However, the identification of vulnerable and less vulnerable functional areas in nursing must be considered a major strength of the study and was only possible by examining the selected items individually. Another strength of the study is that clear implications for specific areas of nursing can be revealed on its data basis, even after adjusting for age, sex, working in a shift system, the treatment of patients with COVID-19, and the impact of COVID-19 on personal life.

### Added value of this study

First, compared with the nurses in other functional areas, the ward nurses exhibited the highest absolute and relative increases in high-intensity stressors, psychological and physical exhaustion, loss of pleasure at work, and intention to leave the profession in the wake of the COVID-19 pandemic. Owing to this finding, this study makes a substantial contribution to the existing literature. In addition, although the OR nurses were exposed to relative increases in high-intensity stressors to the same extent as the IMC nurses, they tended to be more exhausted and more likely intended to leave the profession than the IMC nurses. While the IMC nurses appeared to have adapted to the challenges associated with the COVID-19 pandemic, the OC nurses were the least affected by the impact of the COVID-19 pandemic.

### Nursing implications

The finding that stressors were perceived differently by the nurses across functional areas and had different effects on nurses’ mental and physical health may be relevant to clinical practice in terms of developing preventive and acute intervention strategies. In this context, preventive screening of at-risk nurses using single-item burnout measures could enable the implementation of early supportive intervention strategies tailored to the specific needs of nurses [63], thereby increasing nurses’ organizational and personal-level resilience to COVID-19-associated psychological stress responses [64]. Acute coping strategies, which have already been described in detail [65, 66], should be applied even more specifically to nurses in vulnerable functional areas, namely those in the ward and OR. A reduction in workload [67, 68] and an increase in professional fulfillment [10], which was at a constant level in our collected data regardless of functional area, could represent possible targets for interventions that impose a high level of responsibility on the hospital as an employer [10]. Relating the strong association of workload and nurses’ quality of work life [69] to the findings of our study, new approaches to organizing work schedules and shifts could have significant implications for ward and OR nurses.

### Recommendations for future studies

Future research could benefit from prospective and longitudinal study designs with multiple time points of data collection. In this context, a multicenter study could increase the number of participants. Intervention studies are needed to strengthen the resilience of vulnerable nursing areas in future health system crises. In addition, qualitative studies could further illuminate the causes, interrelationships, and consequences of physical and psychological stressors at the level of nursing.

## Conclusion

Mental health issues appeared to be a constant problem during the acute and non-acute phases of the COVID-19 pandemic. As demonstrated in this study, the impact of the COVID-19 pandemic on nurses’ everyday work may vary across different functional areas. Therefore, coping strategies must be designed to meet the emotional and professional needs of nurses in wards and ORs. Strengthening at-risk nurses’ resilience and retention could provide strategic benefits for prepared health-care systems in the face of future health crises.

## Data Availability

The datasets are available from the corresponding author on reasonable request.

## List of abbreviations

COVID-19: Coronavirus disease
ICU: Intensive care unit
IMC: Intermediate care
MBI: Maslach Burnout Inventory
OC: Outpatient clinic
OR: Operating room
